# Prevalence of respiratory pathogens and risk of developing pneumonia under non-pharmaceutical interventions in Suzhou, China

**DOI:** 10.1017/S0950268823000626

**Published:** 2023-05-02

**Authors:** Ting Shi, Linlin Huang

**Affiliations:** Pediatric Intensive Care Unit, Department of Infectious Diseases, Children’s Hospital of Soochow University, Suzhou, China

**Keywords:** Children, epidemiology, NPIs, pathogens, pneumonia

## Abstract

This study aims to evaluate the impact of non-pharmaceutical interventions (NPIs) on the prevalence of respiratory pathogens among hospitalised children with acute respiratory infections (ARIs) in Suzhou. Children with ARIs admitted to the Children’s Hospital of Soochow University between 1 September 2021 and 31 December 2022 and subjected to 13 respiratory pathogen multiplex PCR assays were included in the study. We retrospectively collected demographic details, results of respiratory pathogen panel tests, and discharge diagnostic information of the participants, and described the age and seasonal distribution of respiratory pathogens and risk factors for developing pneumonia. A total of 10,396 children <16 years of age, including 5,905 males and 4,491 females, were part of the study. The positive rates of the 11 respiratory pathogen assays were 23.3% (human rhinovirus (HRV)), 15.9% (human respiratory syncytial virus (HRSV)), 10.5% (human metapneumovirus (HMPV)), 10.3% (human parainfluenza virus (HPIV)), 8.6% (mycoplasma pneumoniae (MP)), 5.8% (Boca), 3.5% (influenza A (InfA)), 2.9% (influenza B (InfB)), 2.7% (human coronavirus (HCOV)), 2.0% (adenovirus (ADV)), and 0.5% (Ch), respectively. Bocavirus and HPIV detection peaked during the period from September to November (autumn), and MP and HMPV peaked in the months of November and December. The peak of InfA detection was found to be in summer (July and August), whereas the InfB peak was observed to be in winter (December, January, and February). HRSV and HRV predominated in the <3 years age group. HRV and HMPV were common in the 3–6 years group, whereas MP was predominant in the ≥6 years group. MP (odds ratio (OR): 70.068, 95%CI: 32.665–150.298, *P* < 0.01), HMPV (OR: 6.493, 95%CI: 4.802–8.780, *P* < 0.01), Boca (OR: 3.300, 95%CI: 2.186–4.980, *P* < 0.01), and HRSV (OR: 2.649, 95%CI: 2.089–3.358, *P* < 0.01) infections were more likely to develop into pneumonia than the other pathogens. With the use of NPIs, HRV was the most common pathogen in children with ARIs, and MP was more likely to progress to pneumonia than other pathogens.

## Background

Acute respiratory infections (ARIs) are one of the leading types of infectious diseases among children and one of the main reasons for their hospitalisation. Lower respiratory tract illnesses (LRTIs), particularly severe pneumonia, were the major cause of morbidity and mortality in children, especially those under 5 years old [1]. They also imposed a heavy social and economic burden on families. In 2015, pneumonia led to approximately 1 million childhood deaths, mainly in the developing countries [2]. It is evaluated that approximately 21.1 million people suffered from community-acquired pneumonia (CAP) every year in China, second only to India (43.0 million) [3]. A series of pathogens, including viral, bacterial, fungal, and atypical pathogens, accounted for ARIs in children, with viral etiology playing the pivotal role (approximately 80%) [4].

The severe acute respiratory syndrome coronavirus 2 (SARS-CoV-2), shortened to COVID-19, was first discovered in Wuhan, China, in December 2019 [5], and it was listed as a novel respiratory pathogen. To eliminate COVID-19, the Chinese government adopted strict non-pharmaceutical interventions (NPIs), such as banning social gatherings, mandating the wearing of masks, enforcing hand hygiene, sanitising the environment, and delaying the opening of academic institutions and switching to online courses instead [6]. These measures were effective in eliminating the community transmission of COVID-19 in China [7], but the real impact of NPIs on the spread of other respiratory pathogens remains unclear in Suzhou. Ahmed F’s research indicated that social distancing was effective in reducing the spread of seasonal influenza [8]. Studies from multiple countries also observed reductions in respiratory viruses, particularly seasonal influenza (H3N2) and human respiratory syncytial virus (HRSV), under pandemic prevention measures [9–12].

As we all know, RSV, influenza virus, rhinovirus (RV), coronavirus and adenovirus are common pathogens of LRTIs in children [3]. The combined global mortality due to the influenza virus and RSV alone reached 300,000 deaths per year among children under 5 years old [13]. Studies from Shanghai, China, have shown that NPIs helped reduce the spread of influenza and RSV, and had a high incidence of human rhinovirus (HRV) infection during COVID-19 pandemic [14]. In addition, mycoplasma pneumoniae (MP) was recognised as a common cause of CAP, and identified to be responsible for up to 40% of CAP in children older than 5 years of age [15, 16]. But under the impact of NPIs, the prevalence of MP was markedly decreased and showed no obvious seasonality [14]. The effect of NPI implementation on the prevalence of respiratory pathogens among children in Suzhou, China, has not been explored yet. This study will describe the epidemiological characteristics of respiratory pathogens, including HRV, Bocavirus, human parainfluenza virus (HPIV), human coronavirus (HCOV), HRSV, influenza A (InfA), influenza B (InfB), human metapneumovirus (HMPV), adenovirus (ADV), chlamydia (Ch), and MP, in children and explore the high-risk factors of virus-caused pneumonia. It can help clinicians in the timely control of local epidemic pathogens as well as in judging the burden of the disease.

## Participants and methods

### Study site

This study was performed at the Children’s Hospital of Soochow University (SCH), which is the only tertiary children’s hospital in Suzhou, China. It has a capacity of approximately 1,500 beds. There are about 2,281,000 outpatient and emergency visits, and 63,000 hospitalisations at SCH annually.

### Participants

All hospitalised children who were admitted to the Children’s Hospital of Soochow University from 1 September 1 2021 to 31 December 2022 had symptoms of respiratory distress. They were subjected to the 13 respiratory pathogen tests and SARS-CoV-2 DNA PCR assays and were included in the retrospective study. The data, including hospitalisation number, sex, age, diagnosis, and results of the tests conducted, were collected through an electronic case system. A total of 10,396 patients (5,905 males and 4,491 females), with ages ranging from 0 to 16 years, were included in this study. They were divided into four groups by age: <1 year (group I), 1–<3 years (group II), 3–<6 years (group III), and ≥ 6 years (group IV). Based on the climatic conditions of China, the categorisation of the four seasons was done as follows: March, April, and May were considered to be spring; June, July, and August were considered to be summer; September, October, and November were considered to be autumn; and December, January, and February of the next year were considered to be winter. This retrospective study was approved by the Ethics Committee of the Children’s Hospital of Soochow University, China.

### Specimen collection

Nasopharyngeal aspirates were obtained from inpatients within 24 h of their admission. A suction catheter was used to go through the nostrils to the lower portion of the pharynx, and the catheterisation depth was set at 7–9 cm. A total of 2 ml nasopharyngeal aspirates was collected and sent for examination within 30 min.

### Thirteen respiratory pathogens assay by PCR capillary electrophoresis fragment analysis

The 13 respiratory pathogens included HRV, Bocavirus, HPIV, HCOV (Kit untyping assay 229E, NL63, HKU1, and OC43), human syncytial (HRSV), InfA, H1N1 (2009) (H1N1), the seasonal influenza virus A H3N2 (H3N2), InfB, HMPV, adenovirus (ADV), chlamydia (Ch), and mycoplasma (MP).

The nasopharyngeal aspirate was thoroughly mixed and the supernatant aspirated for nucleic acid extraction. 2 μL of the RT-PCR internal reference was added to the specimen, positive control, and negative control, and then mixed with DNA extract (Ningbo Haishi Gene Technology Co., Ltd., Ningbo, China), respectively. Next, 5 μL of the mixture was added to 15 μL of PCR mixture, and centrifuged at 2,000 rpm for 10s. The PCR mixture contained primers specific for highly conserved sequence target regions of the 13 respiratory pathogens (Ningbo Haishi Gene Technology Co., Ltd., Ningbo, China). Real-time PCR was performed on a LightCycler 480II instrument (Roche, Basel, Switzerland) under the following conditions: 25 °C for 5 min, 50 °C for 15 min, and 95 °C for 2 min, followed by 6 cycles of 94 °C for 30s, 65 → 60 °C for 30s, and 72 °C for 60s, and 29 cycles of 94 °C for 30s, 60 °C for 30s, and 72 °C for 60s, finally concluding with 72 °C for 10 min. The fluorescence signal intensity was measured by capillary electrophoresis. All staining procedures were performed according to the manufacturer’s instructions. The results were as follows: amplification products with a site of interest peak height below the low peak for that channel capillary standard were considered negative. Locus of interest peak heights in the amplification product above this channel capillary standard peak were considered positive.

### SARS-CoV-2 DNA PCR assay

Nasopharyngeal swabs were taken from all specimens for Ct value analysis. Detection was performed by RT-PCR with the SARS-CoV-2 nucleic acid detection kit (DaAn Gene Co., Ltd.). Real-time PCR was performed on a LightCycler 480II instrument (Roche, Basel, Switzerland). Values below 5 × 10^2^ copies /ml were considered negative.

### Statistical analysis

Data were presented as number [n(%)] and assayed by chi-square tests. Binary logistic regression analyses were used to calculate the odds ratios (ORs) with 95% confidence intervals (CIs). All statistical analyses were carried out using IBM SPSS Statistics for Windows, version 25 (IBM Corp., Armonk, NY, USA). The GraphPad Prism 9 software was used for mapping. A *P*-value <0.05 was considered statistically different.

## Results

### General Characteristics of enrolled patients

**Figure 1.**
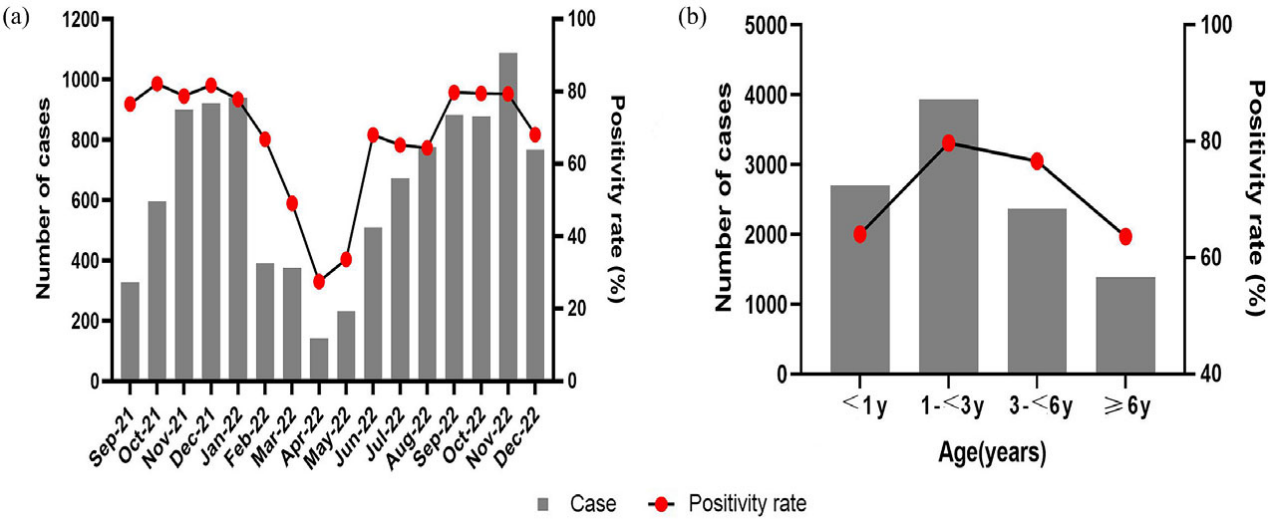
Pathogen detection among children with acute respiratory infections. (a) Monthly distributions of detected pathogens. (b) Distribution of pathogen by age.

A total of 10,396 children <16 years of age, included 5,905 males and 4,491 females, were admitted to SCH between September 2021 and December 2022, and 7,562 (72.7%) were detected positive for the presence of respiratory pathogens. The positive rates of the 11 respiratory pathogen assays were 23.3% (HRV), 15.9% (HRSV), 10.5% (HMPV), 10.3% (HPIV), 8.6% (MP), 5.8% (Boca), 3.5% (InfA), 2.9% (InfB), 2.7% (HCOV), 2.0% (ADV), and 0.5% (Ch), respectively. In addition, the 366 InfA-positive cases contained 358 H3N2 and 3 H1N1. There was no significant difference in gender among the groups with positive pathogen detection, but age showed a statistically significant difference (*P* < 0.01,Table 1). As shown in Figure 1, the number and positive rates of respiratory pathogen detection varied by season and age. The number and positive rate of respiratory virus detection had a trough in summer (March, April, and May). The positive rates of respiratory pathogen detection had a peak in the 1–<3 years group.

**Table 1.** General characteristics of children infected with respiratory pathogens

Parameters	HRV	Boca	MP	HPIV	HCOV	HRSV	InfA	HMPV	ADV	Ch	InfB	*P*
	(*n* = 2,424)	(*n* = 608)	(*n* = 894)	(*n* = 1,072)	(*n* = 280)	(*n* = 1,655)	(*n* = 366)	(*n* = 1,094)	(*n* = 210)	(*n* = 60)	(*n* = 301)	
Sex (male)	1,448 (59.7)	372 (61.2)	474 (53.0)	587 (54.7)	167 (59.6)	934 (56.4)	211 (57.6)	606 (55.4)	125 (59.5)	37 (61.6)	160 (53.2)	0.333
Age (years)												<0.01
<1	513 (21.2)	64 (10.5)	34 (3.8)	271 (25.3)	87 (31.1)	715 (43.2)	36 (9.8)	154 (14.1)	19 (9.0)	52 (86.7)	40 (13.3)	
1–<3	1,009 (41.6)	458 (75.3)	197 (22.0)	537 (50.1)	135 (48.2)	730 (44.1)	109 (29.8)	489 (44.7)	112 (53.3)	6 (10.0)	84 (27.9)	
3–<6	638 (26.3)	79 (13.0)	269 (30.1)	195 (18.2)	43 (15.3)	167 (10.1)	159 (43.4)	406 (37.1)	51 (24.3)	1 (1.7)	102 (33.9)	
≥6	264 (10.9)	7 (1,2)	394 (44.1)	69 (6.4)	15 (5.4)	43 (2.6)	62 (17.0)	45 (4.1)	28 (13.3)	1 (1.6)	75 (24.9)	

*Notes:* The data presented as *n* (%). The chi-square test for categorical variables. Abbreviations: ADA, adenovirus; Ch, chlamydia; HCOV, human coronavirus; HMPV, human metapneumovirus; HPIV, human parainfluenza virus; HRSV, human syncytial; HRV, human rhinovirus; InfA, influenza A; InfB, influenza B; MP, mycoplasma.

### Seasonal and age distribution of various respiratory pathogens

**Figure 2.**
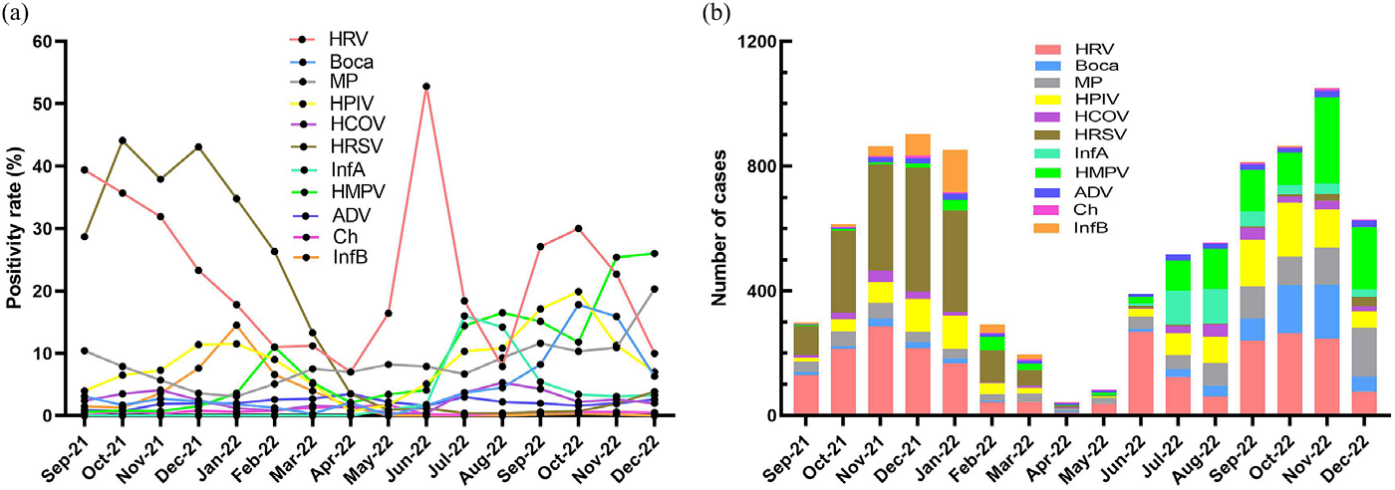
Monthly distributions of 11 respiratory pathogens detection. (a) The positivity of pathogens. (b) The number of pathogens.

**Figure 3.**
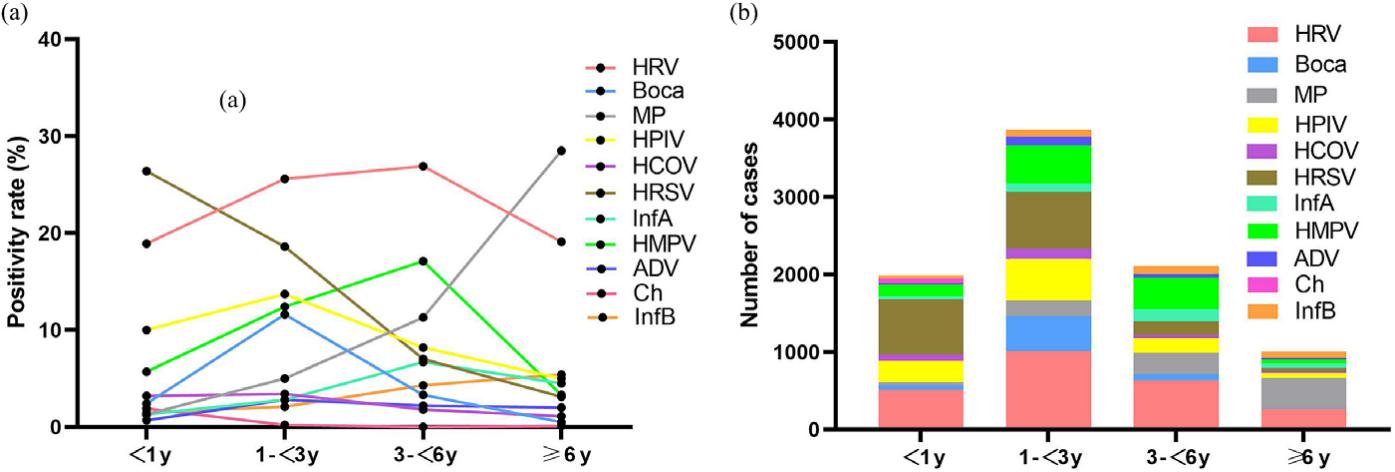
Eleven respiratory pathogens detection among children stratified by age. (a) The positivity of pathogens. (b) The number of pathogens.

As shown in Figure 2, The positivity and number of HRV detections had multiple peaks in various months, but reached the highest in June. The positivity of HRSV peaked in October, November, and December 2021, but did not peak in 2022. The positivity of Bocavirus and HPIV peaked during autumn (September to November). The positivity of MP and HMPV peaked in November and December. The peak of InfA positivity was found to be during summer (July and August), whereas the InfB peak was observed in winter (December, January, and February). HRSV and HRV predominated in the <3 years group (Figure 3). HRV and HMPV were common in the 3–6 years group, whereas MP was predominant in the ≥6 years group.

### Association between season/age and various respiratory pathogens

As shown in Table 2 and Table 3, by multivariate regression analysis, it was found that the HRV infection occurred more often among children aged 3–6 years and in autumn (*P* < 0.01); Bocavirus was commonly found among children aged 1–3 years and also occurred more often in autumn (*P* < 0.01); MP predominated in children aged ≥6 years and majorly in the autumn season (*P* < 0.01); HPIV was commonly found among children aged 1–3 years and was mostly found during autumn (*P* < 0.01); HCOV was mostly found among the children aged <3 years and mainly during summer (*P* < 0.01); HRSV was mostly found among children aged <3 years and majorly found to be in winter (*P* < 0.01); InfA was commonly found among children aged 3–6 years and mainly in summer (*P* < 0.01); HMPV was mostly found among children aged 3–6 years, mainly in summer, autumn and winter (*P* < 0.01); ADV mostly occurred among children aged 1–3 years and in spring (*P* < 0.05); Ch was mostly found among children aged<1 year (*P* < 0.01), mainly in spring; InfB predominated mostly among children aged ≥3 years and in winter (*P* < 0.01).

**Table 2.** Multivariable-adjusted association of age and respiratory pathogen infections

Age (years)	HRV		Boca		MP		HPIV		HCOV		HRSV		InfA		HMPV		ADV		Ch		InfB	
	aOR	*P*	aOR	*P*	aOR	P	aOR	*P*	aOR	*P*	aOR	*P*	aOR	*P*	aOR	*P*	aOR	*P*	aOR	*P*	aOR	*P*
	(95%CI)		(95%CI)		(95%CI)		(95%CI)		(95%CI)		(95%CI)		(95%CI)		(95%CI)		(95%CI)		(95%CI)		(95%CI)	
<1	1		1		1		1		1		1		1		1		1		1		1	
	(reference)		(reference)		(reference)		(reference)		(reference)		(reference)		(reference)		(reference)		(reference)		(reference)		(reference)	
1–<3	1.314	<0.01	4.675	<0.01	3.971	<0.01	1.318	0.001	0.948	0.706	0.676	<0.01	1.766	0.04	2.241	<0.01	4.562	<0.01	0.083	<0.01	2.011	<0.01
	(1.163–1.485)		(3.572–6.119)		(2.747–5.741)		(1.126–1.542)		(0.718–1.251)		(0.597–0.767)		(1.201–2.596)		(1.853–2.710)		(2.787–7.468)		(0.035–0.194)		(1.368–2.956)	
3–<6	1.436	<0.01	1.250	0.194	9.740	<0.01	0.749	0.004	0.493	<0.01	0.215	<0.01	4.333	<0.01	3.245	<0.01	3.392	<0.01	0.023	<0.01	4.238	<0.01
	(1.255–1.643)		(0.893–1.751)		(6.778–13.997)		(0.617–0.911)		(0.340–0.716)		(0.179–0.259)		(2.984–6.290)		(2.667–3.949)		(1.989–5.784)		(0.003–0.169)		(2.906–6.180)	
≥6	1.001	0.988	0.209	<0.01	31.530	<0.01	0.457	<0.01	0.319	<0.01	0.079	<0.01	3.139	<0.01	0.540	<0.01	3.004	<0.01	0.039	0.001	4.123	<0.01
	(0.847–1.183)		(0.095–0.458)		(22.033–45.119)		(0.347–0.600)		(0.183–0.554)		(0.058–0.109)		(2.055–4.797)		(0.385–0.758)		(1.670–5.405)		(0.005–0.282)		(2.775–6.126)	
*p*	<0.01		<0.01		<0.01		<0.01		<0.01		<0.01		<0.01		<0.01		<0.01		<0.01		<0.01	

*Notes:* Model was adjusted for age and season. Abbreviations: ADA, adenovirus; Ch, chlamydia; CI, confidence interval; HCOV, human coronavirus; HMPV, human metapneumovirus; HPIV, human parainfluenza virus; HRSV, human syncytial; HRV, human rhinovirus; InfA, influenza A; InfB, influenza B; Model was adjusted for age and season; MP, mycoplasma; OR, odds ratio.

**Table 3.** Multivariable-adjusted association of season and respiratory pathogen infections

Season	HRV		Boca		MP		HPIV		HCOV		HRSV		InfA		HMPV		ADV		Ch		InfB	
	OR	*P*	OR	*P*	OR	*P*	OR	*P*	OR	*P*	OR	*P*	OR	*P*	OR	*P*	OR	*P*	OR	*P*	OR	*P*
	(95%CI)		(95%CI)		(95%CI)		(95%CI)		(95%CI)		(95%CI)		(95%CI)		(95%CI)		(95%CI)		(95%CI)		(95%CI)	
Spring	1		1		1		1		1		1		1		1		1		1		1	
	(reference)		(reference)		(reference)		(reference)		(reference)		(reference)		(reference)		(reference)		(reference)		(reference)		(reference)	
Summer	2.059	<0.01	5.459	0.001	0.908		3.022	<0.01	3.168	0.001	0.088	<0.01	42.037	<0.01	2.745	<0.01	0.683	0.166	0.301	0.028		0.984
	(1.611–2.632)		(1.978–15.069)		(0.650–1.269)	0.573	(1.953–4.678)		(1.567–6.406)		(0.047–0.166)		(10.406–169.809)		(1.864–4.045)		(0.399–1.171)		(0.103–0.876)			
Autumn	2.861	<0.01	15.233	<0.01	1.247	0.159	3.976	<0.01	2.830	0.003	2.641	<0.01	8.017	0.004	2.370	<0.01	0.485	0.005	0.603	0.173	0.424	0.003
	(2.272–3.603)		(5.658–41.012)		(0.917–1.695)		(2.617–6.040)		(1.432–5.592)		(1.981–3.520)		(1.973–32.578)		(1.630–3.446)		(0.293–0.802)		(0.291–1.248)		(0.243–0.741)	
Winter	1.425	0.04	5.363	0.001	0.912	0.571	3.382	<0.01	1.498	0.267	5.834	<	2.863	0.153	2.314	<0.01	0.742	0.251	0.562	0.130	3.327	<0.01
	(1.120–1.813		(1.959–14.684)		(0.800–1.068)		(2.212–5.171)		(0.734–3.057)		(4.379–7.772)	0.01	(0.676–12.126)		(1.579–3.392)		(0.446–1.235)		(0.267–1.185)		(2.014–5.495)	
*P*	<0.01		<0.01		0.001		<0.01		<0.01		<0.01		<0.01		<0.01		0.011		0.159		<0.01	

*Note:* Model was adjusted for age and gender. Abbreviations: ADA, adenovirus; Ch, chlamydia; CI, confidence interval; HCOV, human coronavirus; HMPV, human metapneumovirus; HPIV, human parainfluenza virus; HRSV, human syncytial; HRV, human rhinovirus; InfA, influenza A; InfB, influenza B; MP, mycoplasma; OR, odds ratio.

### The risk of pneumonia due to various respiratory pathogens

Of the 7,562 children who tested positive for the presence of respiratory pathogens, there were 1,184 with mixed infections, 1,586 with HRV, 326 with Bocavirus, 657 with MP, 793 with HPIV, 156 with HCOV, 1,307 with HRSV, 302 with InfA, 847 with HMPV, 144 with ADV, 35 with Ch, and 225 with InfA (Table 4). By multivariate regression analysis (adjusted for gender, age, and season), MP (OR: 70.068, 95%CI: 32.665–150.298, *P* < 0.01), HMPV (OR: 6.493, 95%CI: 4.802–8.780, *P* < 0.01), Bocavirus (OR: 3.300, 95%CI: 2.186–4.980, *P* < 0.01), HRSV (OR: 2.649, 95%CI: 2.089–3.358, *P* < 0.01), HPIV (OR: 2.193, 95%CI: 1.740–2.764, *P* < 0.01), and InfA (OR: 1.432,95%CI: 1.078–1.903, *P* < 0.013) were more likely to develop pneumonia than the other pathogens.

**Table 4.** The risk of developing pneumonia following infection with various respiratory pathogens

Model	HRV		Boca		MP		HPIV		HCOV		HRSV		InfA		HMPV		ADV		Ch		InfB		*P*
	(*n* = 1,586)		(*n* = 326)		(*n* = 657)		(*n* = 793)		(*n* = 156)		(*n* = 1,307)		(*n* = 302)		(*n* = 847)		(*n* = 144)		(*n* = 35)		(*n* = 225)		
	OR	*P*	OR	*P*	OR	*P*	OR	*P*	OR	*P*	OR	*P*	OR	*P*	OR	*P*	OR	*P*	OR	*P*	OR	*P*	
	((95%CI))		(95%CI)		(95%CI)		(95%CI)		(95%CI)		(95%CI)		(95%CI)		(95%CI)		(95%CI)		(95%CI)		(95%CI)		
Model 1	1		4.355	<0.01	40.238	<0.01	2.547	<0.01	0.835	0.314	4.411	<0.01	0.895		6.373	<0.01	0.723	0.073	4.629	0.011	1.167	0.334	<0.01
	(reference)		(2.911–6.517)		(18.960–85.392)		(2.034–3.190)		(0.587–1.187)		(3.544–5.490)		(0.687–1.165)	0.408	(4.742–8.566)		(0.508–1.031)		(1.411–15.191)		(0.853–1.595)		
Model 2	1		3.300	<0.01	70.068	<0.01	2.193	<0.01	0.724	0.084	2.649	<0.01	1.432	0.013	6.493	<0.01	0.789	0.211	2.972	0.076	1.181	0.345	<0.01
	(reference)		(2.186–4.980)		(32.665–150.298)		(1.740–2.764)		(0.502–1.044)		(2.089–3.358)		(1.078–1.903)		(4.802–8.780)		(0.545–1.144)		(0.893–9.888)		(0.837–1.666)		

*Note:* Model 1 was not adjusted. Model 2 was adjusted for age, gender and season. Abbreviations: ADA, adenovirus; Ch, chlamydia; CI, confidence interval; HCOV, human coronavirus; HMPV, human metapneumovirus; HPIV, human parainfluenza virus; HRSV, human syncytial; HRV, human rhinovirus; InfA, influenza A; InfB, influenza B; MP, mycoplasma; OR, odds ratio.

## Discussion

From early 2020 to December 2022, a series of strict NPIs, including wearing masks, ensuring hand hygiene, delaying the opening of academic institutions, and banning social gatherings, were implemented in China to curb the prevalence of COVID-19 [6]. Under these NPIs, the prevalence of SARS-CoV-2 was rare and infected patients were transferred to designated infectious disease hospitals for treatment. Therefore, the hospitalised children in this study did not include COVID-19 patients. The transmission of SARS-CoV-2 was contained with strict NPIs, but the infection rate of other respiratory pathogens in ARI (72.7%) has not decreased, contrary to the results of the study in Shanghai (27.5%) [14]. In the study, HRV instead of RSV was found to be the dominant pathogen in children with ARIs. In addition, MP infection was more likely to progress to pneumonia than the other pathogens. It implied that the implementation of NPIs may have affected the prevalence and pathogenicity of common respiratory pathogens.

The finding from this study that the common respiratory pathogens were prevalent in winter and spring [17] was consistent with the findings of previous studies. However, these were mostly observed among children aged 1–3 years, which may be because of young children not complying with the NPIs and indulging in outdoor activities. RSV is a seasonal virus and its epidemiology varies with geographic region and climate [17]. Suzhou belongs to the subtropical region of the Northern Hemisphere, and virus diffusion usually occurs between October, November, and March, with peak incidence in January and February [18]. In the study, the prevalence peak of RSV appeared in September–December 2021, but not in 2022. This must be due to virus–virus competition and interference [19]. In addition, RSV tended to circulate in infants and indicated a decreasing trend with increasing age, which was consistent with the results of previous studies [20]. It implied that NPIs did not appreciably affect RSV seasons and population characteristics, but also does not rule out an association of greater enforcement of NPIs with increasing age.

HRV instead of RSV emerged as the most detectable respiratory pathogen in this study, which was consistent with previous studies [14, 21, 22]. The reasons were that HRV was a non-enveloped virus, which was relatively resistant to ethanol-containing disinfectants [23], and that it could survive for a prolonged period on environmental surfaces [24]. In this study, HRV was prevalent in summer and autumn and was mostly observed among children aged 1–6 years. However, the studies by Jiang et al. indicated that HRV had a high prevalence from March to September (spring and summer) and was common in all age groups before the COVID-19 epidemic [25]. A possible reason for this division was the lack of fit of NPIs among children aged 1–6 years.

Before the COVID-19 epidemic, the detection rate of influenza virus in the center (SCH) was 11.3%–16.9% and the dominant strains were H3N2 and InfB [26, 27]. Under the implementation of NPIs, the dominant strains of influenza virus have not changed, but the detection rate has shown a marked decline (6.4%). It was also observed in many other areas such as Shanghai, Hong Kong, and New Zealand [9, 14, 28]. The influenza virus had an obvious seasonal pattern in Suzhou, and InfA prevailed mainly in summer and InfB in winter, which is consistent with previous studies [27]. In addition, the older children in the group (≥3 years) were more susceptible to the influenza virus, which may be due to a variety of factors, such as influenza vaccination, inevitable outdoor activities, etc.

Remarkably, the prevalence of HPIV (10.3%) and HMPV (10.5%) had an obvious increase in ARIs compared to previous studies in Suzhou (4.0%–4.8% and 3.5%) [25, 27]. In this study, the positivity of HPIV peaked in the 1–3 years group and HMPV in the 3–6 years group, which was consistent with previous studies [27]. Unlike previous reports, the seasonality of HPIV and HMPV circulation was found to have changed. The peak of HPIV and HMPV positivity was not observed in the spring season. Additionally, the epidemic season of Bocavirus also changed from summer to autumn. In addition to NPIs, this may be due to other factors such as viral competition, immune response through viral proteins, and interference.

Mycoplasma, as one of the common respiratory pathogens, was detected in 8.6% of pediatric inpatients in this study, which was lower than what was suggested by reports prior to the COVID-19 epidemic (15.8%) [25]. MP infection usually occurred in winter and also happened round the year, which was consistent with the seasonal pattern in this study. The positivity of MP in the ≥6 years age group was found to be the highest, which was similar to the study by Waites KB et al. [16]. MP was transmitted from person to person via respiratory droplets during close contact, which may have been reduced by the implementation of NPIs. But their seasonal and age characteristics have not changed.

Under the enforcement of NPIs, the epidemiological characteristics of respiratory pathogens have changed, and the disease burden of them contributing to childhood pneumonia has diverged. Ashley C’s study indicated that the most common pathogen leading to CAP in children was HRV, followed by influenza, *Streptococcus pneumoniae*, and MP [29]. In this study, MP, HMPV, and Bocavirus infections were more likely to progress to pneumonia than other pathogens in children. Therefore, we speculate that MP, HMPV, and Boca in children should be more carefully monitored in the future.

## Conclusion

With the implementation of NPIs, HRV replaced HRSV as the most common pathogen implicated in children with ARIs. HPIV and HMPV, as common respiratory pathogens, have changed their endemicity seasons; The positivity of MP and influenza viruses was low, but their epidemic season and age of occurrence were found to have changed. In addition, MP, HMPV, and Boca infections were more likely to progress to pneumonia than other pathogens under enforcement of NPIs in children.

Our study is limited. Firstly, bacterial infection was not considered in this study. Second, the observation period was not long enough, and further observational studies are needed to help us deal better with ARIs.

## Data Availability

The data used in this study are available from the corresponding author upon reasonable request.

## Abbreviations

ADV: adenovirus
ARIs: acute respiratory infections
CAP: community-acquired pneumonia
Ch: chlamydia
CIs: confidence intervals
HCOV: human coronavirus
HMPV: human metapneumovirus
HPIV: human parainfluenza virus
HRSV: human respiratory syncytial virus
InfA: influenza A
InfB: influenza B
LRTIs: lower respiratory tract illnesses
MP: mycoplasma pneumoniae
NPIs: non-pharmaceutical interventions
ORs: odds ratios
RV: rhinovirus
SARS-CoV-2: severe acute respiratory syndrome coronavirus 2
